# Salt stress affects mRNA editing in soybean chloroplasts

**DOI:** 10.1590/1678-4685-GMB-2016-0055

**Published:** 2017-03-02

**Authors:** Nureyev F. Rodrigues, Guilherme C. da Fonseca, Franceli R. Kulcheski, Rogério Margis

**Affiliations:** 1 Departamento de Genética, PPGBM, Universidade Federal do Rio Grande do Sul (UFRGS), Porto Alegre, RS, Brazil; 2 Centro de Biotecnologia, PPGBCM, Universidade Federal do Rio Grande do Sul (UFRGS), Porto Alegre, RS, Brazil; 3 Departamento de Biofísica, Universidade Federal do Rio Grande do Sul (UFRGS), Porto Alegre, RS, Brazil

**Keywords:** small RNA, chloroplast, RNA editing, PPR, salt stress

## Abstract

Soybean, a crop known by its economic and nutritional importance, has been the subject of several studies that assess the impact and the effective plant responses to abiotic stresses. Salt stress is one of the main environmental stresses and negatively impacts crop growth and yield. In this work, the RNA editing process in the chloroplast of soybean plants was evaluated in response to a salt stress. Bioinformatics approach using sRNA and mRNA libraries were employed to detect specific sites showing differences in editing efficiency. RT-qPCR was used to measure editing efficiency at selected sites. We observed that transcripts of *NDHA*, *NDHB*, *RPS14* and *RPS16* genes presented differences in coverage and editing rates between control and salt-treated libraries. RT-qPCR assays demonstrated an increase in editing efficiency of selected genes. The salt stress enhanced the RNA editing process in transcripts, indicating responses to components of the electron transfer chain, photosystem and translation complexes. These increases can be a response to keep the homeostasis of chloroplast protein functions in response to salt stress.

## Introduction

Soybean (*Glycine max* L.) is one of the major legume crops in the world, providing an abundant source of oil and protein-rich food for human and animal consumption (Le *et al*., 2012). The high demand for protein in meals drove to further expansion of oilseed production and has favored an increase of soybean production, especially in Brazil (Guevara *et al*., 2015). In Brazilian agriculture, soybean is the most important crop. Currently, Brazil is the second largest producer behind the United States. Soybeans are expected to continue being the most lucrative export product with more than half of Brazilian production destined for world markets (Guevara *et al*., 2015). However, like many crops, soybean is subject to several abiotic stresses that reduce its yield.

Plants are exposed to a range of stress conditions such as oxidative stress, variant temperature, light intensity, waterlogging, drought and salinity. These abiotic stresses affect the whole plant, compromising basic molecular and physiological aspects from germination to the reproduction phases (Mahajan and Tuteja, 2005). Salt stress is one of the main environmental stresses, and it affects economically important crop species that are very sensitive to salinity, such as bean (*Phaseolus vulgaris*), maize (*Zea mays*), rice (*Oryza sativa*) and soybean (Wang *et al*., 2003; Zheng *et al*., 2009). Salt-affected soils occur in more than 100 countries and their worldwide extent is estimated at about 1 billion ha (FAO and ITPS, 2015). Salinity stress affects mainly lipids, ions levels, malate and nitrogen metabolism, anti-oxidative enzymes and antioxidants, chloroplast structure and photosynthesis (Parida and Das, 2005). Many studies have been dedicated to the impact of salinity on photosynthetic activity, carbon assimilation, pigment composition, electron transport, and photosystem I and II efficiency (Sudhir *et al*., 2005; Parida and Das, 2005; Koyro, 2006). Clearly, there is a link between effects on photosynthesis and chloroplast, however, certain works have looked specifically at plastid salt stress effects (Gomez, 2003; Zhang *et al*., 2008; Zheng *et al*., 2009).

Chloroplasts are complex organelles that have their own gene expression machinery, intricate post-transcriptional processes and a fine coordination with nuclear gene expression. Chloroplasts have received particular interest because they are responsible for photosynthesis. Alterations in metabolic pathways, in specific signals like redox state, or in protein structures can lead to disruption in plastid activity and, consecutively, in plant yield. RNA editing, a post transcriptional process, consists in nucleotide conversions from cytosine (C) to uracil (U), or, less frequently, from U to C. This process, also present in mitochondria, is performed by deamination and amination reactions (Chateigner-Boutin and Small, 2011; Hayes *et al*., 2015). Usually, editing events preserve amino acids that are phylogenetically conserved by restoring the codon sequence. The most frequent change is serine to leucine, but other alterations, including silent or non-conservative changes, have also been described (Inada *et al*., 2004; Chateigner-Boutin and Small, 2010). In both organelles, editing can create an initiation codon, and create or remove stop codons. Editing can also be found in introns (prerequisite for splicing in some cases) and in untranslated regions (UTR) (Takenaka *et al*., 2008; Castandet and Araya, 2011). This powerful and intriguing process has been studied due its essential function and also because of the impact in the evolutionary process (Takenaka *et al*., 2013).

Plastid RNA editing depends on the editosome machinery to precisely process the emerging transcripts. The editosome composition has not yet been completely identified. However, some components of the editing machinery, like the pentatricopeptide repeat (PPR) proteins, were already recognized. The PPR motif is a 35-amino-acid repeat that folds into a pair of antiparallel alpha helices. Arrays of tandem PPR motifs form a superhelical ribbon-like sheet (Small and Peeters, 2000; Barkan and Small 2014). In land plants, the PPR gene family contains from 400 to more than 1000 members (Barkan and Small, 2014). The PPR proteins are classified into two major subfamilies, P-type and PLS-type PPRs. The PLS-type PPR proteins can be further divided into three subgroups: E, E+, and DYW, that differ in the presence of an optional C-terminal region (Lurin *et al*., 2004). Most PLS-type PPR proteins involved in editing act as site-recognition factors, recognizing the 5′ region upstream of the editable C residue (Yagi *et al*., 2013). PLS-type PPR proteins presenting cytidine deaminase motifs within the DYW domain have been described as being directly responsible for RNA editing activity (Boussardon *et al*., 2014; Wagoner *et al*., 2015). Other PPR proteins, as HCF152 and PPR10, are involved in intercistronic processing of polycistronic precursor transcripts or in stabilizing specific RNAs (Barkan and Small, 2014; Yap *et al*., 2015).

Diverse studies have been done to analyze editing regulation of plastids under various situations, such as tissue-specific differences, responses to molecular signals, effects in immunity, and responses to abiotic stress (Kakizaki *et al*., 2012; García-Andrade *et al*., 2013; Tseng *et al*., 2013). The potential of the RNA editing efficiency as a marker for stress tolerance or as a target for genetic modification was evaluated in some studies. For example, incomplete editing caused by increased temperature is correlated with change in plastid translation in maize (Nakajima and Mulligan, 2001). Specifically, heat stress leads to loss of editing sites and intron splicing reactions in *NDHB* transcripts (Karcher and Bock 2002). Variations in the efficiency of plastid editing in *NDH* transcripts was evaluated and not linked to differences in drought tolerance in perennial ryegrass (*Lolium perenne*) (Van Den Bekerom *et al*., 2013).

Most of the studies on RNA editing have used the reverse transcription PCR (RT-PCR) method of total chloroplast mRNAs and cloning of several chloroplast cDNA fragments into vectors to be sequenced (Rüdinger *et al*., 2009). Another method is to design primers to amplify target genes from cDNA samples and sequence them (Wolf *et al*., 2004). RNA editing events could also be detected by using chloroplast cDNA datasets as templates for amplification in Poisoned Primer Extension methodology, or also by High Resolution Melting (HRM) analysis (Chateigner-Boutin and Small, 2007). Many plastid small RNAs (sRNAs) showed sequence similarities to PPR-binding sites, which provides support to the idea that large amounts of sRNAs remnants resulted from PPR protein targets (Ruwe and Schmitz-Linneweber, 2012). In this way, several chloroplast sRNAs are recovered as RNA-binding protein footprints, including PPR-editosome components, which remain in the sequencing results due to protein protection against ribonucleases.

Despite several different methodologies already described in the literature for RNA-editing recognition, in this work we evaluated the impact of salt stress on soybean C to T editing efficiency by a new method comprised by *in silico* screening of editing sequences of sRNA libraries obtained by high-throughput sequencing, followed by RT-qPCR assays.

## Materials and Methods

### Plant material, stress treatment and RNA isolation

Soybean plants were grown over 8 days using Hoagland solution. After this period, six plants were transferred into a new Hoagland solution (establishing the control group), and six plants were submitted to a salt-stress treatment using a Hoagland solution supplemented with 200 mM NaCl. Leaves were collected after intervals of 4 and 24 hours and stored in liquid nitrogen until RNA extraction. Total RNA from leaves was isolated using TRIzol reagent (Invitrogen, CA, USA), and the RNA quality was evaluated by Nanodrop quantification and gel inspection.

#### sRNA/mRNA libraries, chloroplast genome, and prediction of conserved editing sites

Public sRNAs and mRNAs libraries of *G. max* leaves, deposited in NCBI GEO (http://www.ncbi.nlm.nih.gov/geo/), accession number GSE69571, were used in this study to evaluate the differential RNA editing rate when exposed to saline stress. Complete chloroplast genome and coding sequences, as well as tRNAs from soybean (NC_007942) were obtained separately from the Index of Genomes from the Chloroplast Genome Database (http://chloroplast.ocean.washington.edu/). To predict editing sites and evaluate their editing rates, the PREP-Cp tool (http://prep.unl.edu/) (Mower 2009) was used with a cutoff value of 0.5, in spite of the 0.8 default value, using the coding sequences of the chloroplast genome mentioned above.

### Analyses of edited sRNAs

The sRNAs libraries were primarily aligned against the chloroplast genome, coding sequences and tRNAs, using Bowtie software (Langmead *et al*., 2009) with 0 mismatch and not allowing reverse complement matches. The aligned reads resulted in a new file called cp_m0. The unaligned reads were submitted to a second round of alignment with 0 mismatch, against nuclear and mitochondrial genomes. The unaligned reads were further aligned with two mismatches, and no reverse complement matches were allowed against the chloroplast genome and coding sequences. This second group of aligned reads produced another file called cp_m2. Both cp_DNA fastq files were concatenated in a cp_m0_m2 file. The cp_m0_m2 files were aligned against chloroplast coding sequences using Geneious (Kearse *et al*., 2012) R8 with the Bowtie algorithm, using the same parameters of the previous alignments. The Geneious Find Variation/SNPs tool was used with parameters set as follows: Minimum Coverage of 5, Maximum Variant P-Value of 10^-2^, to find polymorphism Inside and Outside coding sequence and P-value calculation method as approximate. The coverage values of edited and non-edited reads were transposed to the implementation of statistical analysis. The same pipeline was used to analyze editing rates with mRNA data.

### Differential expression analysis

SAM files created in the bowtie alignment were utilized to generate a count table containing data from all libraries. This table was the input file to differential expression analysis performed using DeSeq2 package (Anders and Huber, 2010) implemented in R package (R Core Team, 2015). Heatmaps were generated with normalized counts of all plastid genes for data visualization.

### Editing analysis by RT-qPCR

The cDNA synthesis was carried out using approximately 1 μg of total RNA. The d26T primer was used in each reaction. Before transcription, RNA and primers were mixed with RNase-free water to a total volume of 10 μL and incubated at 70 °C for 5 min followed by ice-cooling. Then, 3 μL of 5 RT-Buffer (Promega, Madison, WI, USA), 1 μL of 5 mM dNTP (Ludwig, Porto Alegre, RS, Brazil) and 1 μL of MMLV-RT Enzyme 200 U (Promega, Madison, WI, USA) were added for a final volume of 20 μL. The synthesis was performed at 42 °C for 30 min in a Veriti Thermal Cycler (Applied Biosystems, Foster City, CA, USA), and inactivation of the enzyme was completed at 85 °C for 5 min. All cDNA samples were 100-fold diluted with RNase-free water before being used as a template in RT-qPCR analysis.

**Figure 1:**
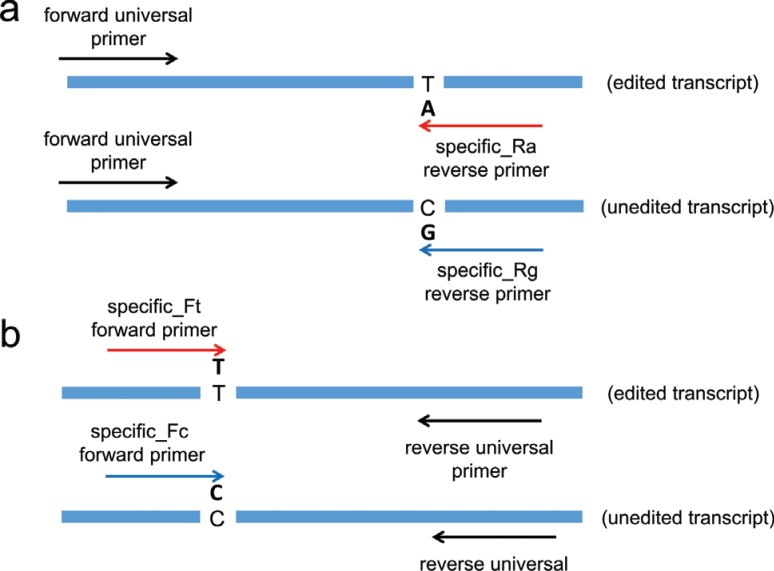
Schematic illustration of qPCR analysis of RNA editing frequency showing relative locations of (A) specific-reverse and (B) specific-forward qPCR primers. Arrows depict the annealing sites of qPCR primers.

A set of primers was designed according to (Chen *et al*., 2008) with modifications. For each editing site, we designed a set of primers composed by two specific editing primers and one unique universal primer. When the specific editing primers were designed as forward, the universal primer was designed as reverse and vice-versa. The specific editing primers containing a unique difference in the first nucleotide recognized the edited or unedited site (Figure 1). The RT-qPCR reactions were performed in a Bio-Rad CFX384 real time PCR detection system (Bio-Rad, Hercules, CA, USA) using SYBR Green I (Invitrogen, Carlsbad, CA, USA) to detect double-stranded cDNA synthesis. Reactions were completed in a volume of 10 μL containing 5 μL of diluted cDNA (1:100), 1 SYBR Green I (Invitrogen, CA, USA), 0.025 mM dNTP, 1 PCR Buffer, 3 mM MgCl2, 0.25 U Platinum Taq DNA Polymerase (Invitrogen, CA, USA) and 200 nM of each universal and C or T-specific primer set. Samples were analyzed in technical quadruplicate in a 384-well plate, and a no-template control was included. The conditions were set as follows: an initial polymerase activation step for 5 min at 95 °C, 40 cycles for 15 s at 95 °C for denaturation, 10 s at 60 °C for annealing and 10 s at 72 °C for elongation. A melting curve analysis was programmed at the end of the PCR run over the range of 65 to 99 °C, and the temperature increased stepwise by 0.5 °C.

Threshold and baselines were manually determined using the Bio-Rad CFX manager software. To calculate the relative expression of transcripts we used the 2^-ΔΔCt^ method (Livak and Schmittgen, 2001). Primer efficiencies were calculated by LinRegPCR software (Ruijter *et al*., 2009) to evaluate a possible amplification by primer efficiency bias. By doing so we obtained independent estimates of amplification efficiency for each primer in each treatment. Differences in plastid transcript editing among treatments were detected using two-tailed Student's *t*-tests between means. Significance was set at p < 0.05. Tests were performed with R package software (R Core Team, 2015).

## Results

### Rates of editing in sRNAs libraries

The PREP analysis carried out on soybean chloroplasts identified 20 different genes that contained RNA editing sites (Table S1). All predicted editing sites were confronted with the aligned sRNA reads in order to evaluate the presence/absence of editing events. Edited reads were identified in a set of 16 genes from at least one of the sRNAs library (Table 1). Among 87 predicted edited sites, 34 were confirmed by sRNAs reads. Other predicted sites, even with a higher PREP score value, that should indicate a higher confidence, could not be confirmed because they did not present enough coverage (Table S1). A group of four genes was selected considering their total coverage and for being sites with statistical differential values of edited reads between control and salt treatment: *NDHA*-1073 (p = 0.033), *NDHB*-149 (p = 0.046), *RPS14*-80 (p = 0.079) and *RPS16*-212 (p = 0.073) (Table 1). Other editing sites showed relevant p-value in leaves libraries, however, they were not selected when their total coverage was lower than four reads (Table 1).

**Table 1:** Quantitative distribution of sRNAs reads in plastid editing sites, editing percentages and p-values (t-test).

Gene	Position (nt)	PREP score	Cnt-1	% edition	Cnt-2	% edition	Salt-1	% edition	Salt-2	% edition	p-value
***NDHA***	**1073**	**1**	**4**	**0.75**	**1**	**1**	**1**	**0.20**	**9**	**0.60**	**0.033**
***NDHB***	**149**	**1**	**11**	**0.55**	**4**	**0.80**	**6**	**0.33**	**5**	**0.36**	**0.046**
***PSBF***	**77**	**1**	**8**	**1**	**10**	**1**	**14**	**1**	**7**	**1**	**-**
***RPS14***	**80**	**1**	**24**	**0.75**	**17**	**0.85**	**14**	**0.88**	**19**	**0.90**	**0.079**
***RPS16***	**212**	**0.83**	**10**	**0.90**	**6**	**0.75**	**4**	**0.57**	**9**	**0.75**	**0.073**
*ACCD*	617	0.8	7	0.86	5	1	8	1	0	nd	0.275
*ATPF*	92	0.86	0	nd	3	1	3	1	3	1	0.225
*CLPP*	559	1	16	0.81	13	0.81	8	1	10	0.71	0.643
*MATK*	935	0.57	6	ne	0	nd	0	nd	1	0.08	0.225
*NDHB*	542	1	0	nd	1	1	1	1	0	nd	1.00
	586	1	0	ne	1	1	2	1	0	nd	1.00
	737	1	1	1	2	1	0	nd	0	nd	-
	746	1	1	1	4	1	0	nd	1	0.50	0.035
	830	1	0	ne	1	0.50	1	1	4	0.67	0.035
	836	1	0	ne	2	1	0	nd	6	0.86	0.860
	1112	1	6	0.67	4	1	5	0.83	3	0.60	0.383
	1255	1	1	1	0	nd	0	nd	0	nd	0.225
	1481	1	3	1	3	1	2	0.67	4	1	0.225
*NDHD*	2	1	1	ne	1	1	0	nd	0	nd	0.225
	674	1	0	nd	0	nd	1	1	0	nd	0.225
	878	1	1	ne	2	0.67	2	1	2	0.67	0.104
	1298	0.8	0	nd	2	1	0	nd	0	nd	0.225
*NDHF*	586	0.8	1	ne	1	0.33	0	nd	0	nd	0.225
*PSAI*	79	1	0	nd	1	1	3	1	0	nd	1.000
*PSBE*	214	1	23	0.91	20	0.91	20	0.91	24	1	0.239
*RPOB*	338	1	2	0.50	1	1	0	nd	1	1	0.496
	551	1	0	nd	1	1	0	nd	0	nd	0.225
	566	1	0	nd	1	0.33	0	nd	1	0.50	0.660
	2000	1	1	1	0	nd	1	1.00	1	0.20	0.801
	2819	1	2	0.50	0	nd	0	nd	0	nd	0.225
*RPOC1*	41	1	0	nd	1	1	0	nd	0	nd	0.225
	488	0.71	0	nd	0	nd	0	nd	2	0.67	0.225
*RPOC2*	3284	0.57	2	0.50	0	nd	0	nd	0	nd	0.225
*RPS14*	194	0.71	20	0.05	26	0.04	9	0.11	11	0.09	0.003

Ne: no edition; nd: not defined (without coverage)

Specific primers were designed to detect edition in the four genes and also in *PSBF*-77 (Table S2) that presented 100% of edited reads in all anchored sRNAs. Except for *RPS14*-80, sRNA analysis demonstrated that in the selected genes, the editing percentage was higher in control libraries than in salt-treated ones (Table 1). A parallel analysis of editing sites using mRNA data showed relevant values in coverage and edited reads that shared similar patterns to those observed with sRNA, except for *NDHA*-1073 and *NDHB*-149 (Table S3).

### Rate of editing of chloroplast transcripts by RT-qPCR

RT-qPCR was used to measure the relative amount of edited and unedited plastid transcripts at 4 and 24 hours, comparing control and salt treatment. Using LinRegPCR software, the efficiency of each amplification was calculated; for each editing primer, only reactions with efficiency higher than 1.75 were maintained in the analysis. The mean efficiency of all primers was higher than 1.80, and was not significantly different when compared with the pairs of C/G and T/A specific primers (Table S4).

**Figure 2:**
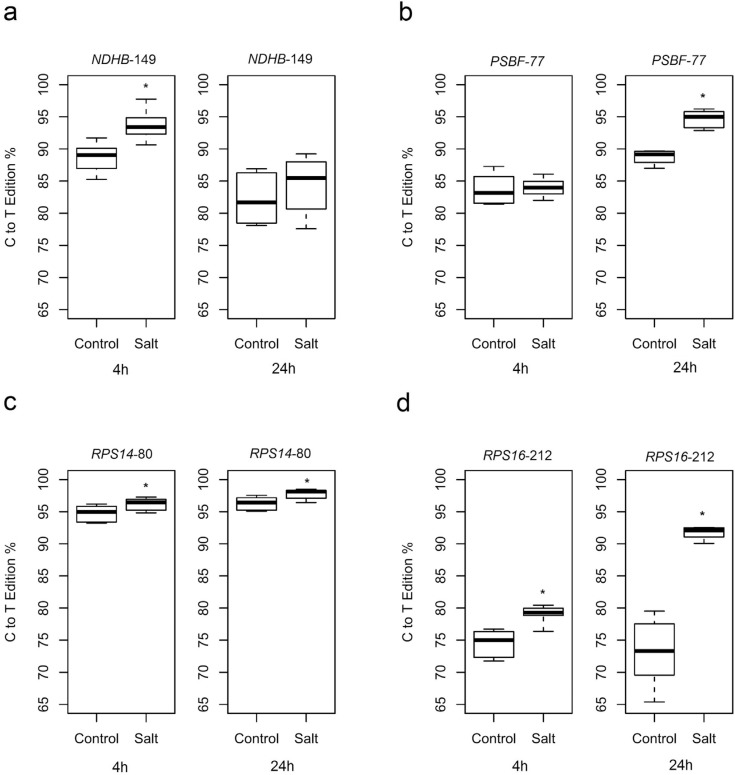
Boxplot indicating the editing of (a) *NDHB*-149, (b) *PSBF*-77, (c) *RPS14*-80, and (d) *RPS16*-212 sites of control and salt stress plants, in 4h and 24 hours treatment. Box area represents the lower and the upper percentiles. The upper whisker of the boxplot indicates the highest editing value observed; the lower whisker, the lowest editing value; and the middle line, the median editing value. Asterisk indicate significantly different values at P < 0.05.

The rate of edition was affected in all four genes when leaf samples were collected 4 hours after the salt treatment. The percentage of C to T editing varied in all genes. A statistically significant increase in RNA edition was observed for salt-treated samples: *NDHB-*149 presented an increase in editing from 88.7% to 93.7% (p = 0.004) (Figure 2a), *RPS14*-80 from 94.76% to 96.20% (p = 0.05) (Figure 2c) and *RPS16*-212 from 74.5% to 78.99% (p = 0.003) (Figure 2d). *NDHA*-1073 presented an absolute reduction in the average of editing percentage, but due to variance, without statistical significance (from 77.79% to 70.53%, p = 0.285) (Figure S3); the *PSBF*-77 editing percentage was not significantly different (from 83.36% to 84%, p = 0.629) (Figure 2b). When salt treatment was extended to 24 hours, an increase in editing percentage was verified in *PSBF*-77 from 88.75% to 94.70% (p = 0.0001) (Figure 2b), *RPS14*-80 from 96.31% to 97.76% (p = 0.025) (Figure 2c) and *RPS16*-212 from 73.10% to 91.65% (p = 0.0002) (Figure 2d). *NDHA*-1073 and *NDHB*-149 presented no statistical differences in their editing percentages, with values from 61.51% to 60.97% (p = 0.861) (Figure S3), and from 82.18% to 84.39% (p = 0.395) (Figure 2a) respectively.

In order to evaluate if differences in editing efficiency could be correlated with transcriptional rate, a differential gene expression of chloroplast editing genes was performed using RNA sequence libraries. In sRNAs libraries, no differences were found between control and salt treatment for the analyzed chloroplast editing genes (Figure S1). The same analysis of chloroplast gene expression was performed with mRNA libraries, and no differences were found (Figure S2a). Contrarily, when all nuclear genes were compared, a differential expression was detected.

## Discussion

Plant responses to salt stress have been examined due to their agronomic implications. Our results demonstrated variability in plastid transcript editing in soybeans, in response to salt treatment. The selected editing sites showed different coverage of sRNAs when control samples were compared to salt treated ones. Plastid sRNAs present as peaks of sequence reads indicated that they are found at coverage levels similar to, or even higher than matching mRNAs (Zhelyazkova *et al*., 2011). The parameters that determine the rate of the initiating endonucleolytic cleavage for chloroplast RNA decay are not known. These parameters are likely to include sequence and structure of mRNAs, their extent of ribosome association, and the presence of other RNA-binding proteins that mask or expose potential RNase cleavage sites (Barkan, 2011). Therefore, an increase in translation and consequent protection by the ribosome and PPR-like proteins association can lead to a reduction in the degradation of edited transcripts. This could explain the reverse correlation between total sRNA coverage decrease in editing sites and the increase in editing percentage demonstrated by RT-qPCR assays, as observed for *NDHB*-149.

The *NDHB* gene encodes part of the hydrophobic thylakoid-inserted arm in the NAD(P)H dehydrogenase (NDH) complex; this complex plays a role in alleviating over-reduction in the stroma under stress conditions (Martín and Sabater, 2010; Peng *et al*., 2011); therefore, the increase in *NDHB*-149 editing found after 4 hours of salt treatment could contribute to the maintenance of the NDH complex, avoiding an initial impact in the redox state of plastids in treated plants. Moreover, *NDHB* editing maintenance is also essential to cyclic electron flow around photosystem 1 (CEF1), that has been demonstrated as a correlated process in salt tolerance (Lu *et al*., 2008). In *G. max* varieties, chlorophyll fluorescence, NDH-dependent CEF activity, *NDHB* mRNA abundance, and constitutive levels of NDH-B protein were much higher in a salt-tolerant variety than in the salt-sensitive one (He *et al*., 2015);. The elevated editing percentage, observed 4 hours after salt treatment, can be linked to this increase in translation of the *NDHB* gene and NDH-dependent CEF activity enhancement in the salt-tolerance response. Our chloroplast gene expression data presented no differences, but other experimental approaches are necessary to confirm a possible role of transcriptional changes in the increase of editing. After 24 hours of treatment, the *NDHB* editing level returned to normal baseline, possibly causing a mechanism by which the photosynthesis system can be impaired, when ROS begin to cause effects, such as inhibition of PSII repair and of protein synthesis.

The impact of non-editing of the *PSBF* plastid gene has been described in an *LPA66* mutant for which a PPR responsible for editing *PSBF*-77 should be encoded. Its morphological aspects were reduced growth, and pale green leaves under optimal growth, due to perturbed PSII functions (Cai *et al*., 2009). In our results, the editing percentage of *PSBF*-77 showed an increase during the salt stressed condition, probably aiming at translation and repair enhancement of PSII. Although after 24 hours of treatment an increase in editing percentage of *PSBF* transcripts (component of PSII) occurred, salt stress has been reported to enhance photodamage to PSII by excess ROS suppressing transcription and translation of the *PSBA* gene and inhibiting the repair of PSII in *Synechocystis* (Kreslavski *et al*., 2007; Murata *et al*., 2007).

The *RPS14* and *RPS16* genes encode small ribosomal subunits, and among the plastid ribosomal genes, *RPS16* is an essential plastid gene that cannot be inactivated, having thus, an important role in the translation process (Tiller *et al*., 2012). In both treatment intervals, the editing percentage showed an increase, being higher at 24 hours than at 4 hours of treatment. This increase can be related to a need for further translation of plastid proteins under salt stress. Decreased or incomplete editing of *RPS14* and *RPS16* transcripts can affect the plastid-encoded protein synthesis. Effects of incomplete editing in *RPS12* were reported, resulting in the synthesis of polymorphic polypeptides in plant mitochondria (Phreaner, 1996). In heat stress, the editing status of *RPS14* decreased rapidly in response to change in temperature, and it remained low after an extended period of acclimatization (Nakajima and Mulligan, 2001). *RPS14* and *RPS16* gene expression is regulated by cytokinins (CK) and abscisic acid (ABA) (Cherepneva *et al*., 2003; Yamburenko *et al*., 2013). Chloroplast transcription can be stimulated by CK in response to ABA, drought, and salt-induced senescence. Specific ABA and stress-responsive CK receptors have been described, and maybe a cross-talk among CK, ABA and stress signaling pathways exists (Tran *et al*., 2007). The increase in editing of *RPS14* and *RPS16* transcripts can be linked to a CK response against salt-induced senescence.

Based on our results, salt stress enhances the editing process in transcript components of the NDH, PSII, and translation complexes. All analyzed editing sites had a percentage of increase that can be a response to keep homeostasis of chloroplast functions. The maintenance of edited codons seems to be essential for protein function, and the editing process responds to this demand. Other studies that measure transcription, editing and translation of edited genes in different time intervals and salt concentrations can help to reveal the floating diversity in all edited transcripts and correlate these to other salt stress-induced responses of the editing process.

## Supplementary Material

The following online material is available for this article:

Table S1 - Editing analyses of plastid CDS from small RNA seq.

Table S2 - Sequences and descriptions of real time primers.

Table S3 - Editing analyses of plastid CDS from mRNA seq.

Table S4 - Means of RT-qPCR primer efficiency and correlation.

Table S5 - Identification of chloroplast genes in heatmap.

Figure S1 - Heatmap of relative expression of plastid genes.

Figure S2 - Heatmap of relative expression of plastid genes and differentially expressed nuclear genes.

Figure S3 - Boxplot of percentage editing of the NDHA-1073 editing site.
