# Supplementary material for: Salt stress affects mRNA editing in soybean chloroplasts
Source: Genet Mol Biol. 2017 Mar 2;40(1 Suppl 1):200–8. doi: 10.1590/1678-4685-GMB-2016-0055 (PMC5452132; doi:10.1590/1678-4685-GMB-2016-0055)
Supplement: Supplementary file 1 [file 1415-4757-gmb-1678-4685-GMB-2016-0055-Suppl01.pdf]

Table S1- Editing analyses of plastid CDS using PREP prediction and reads derived from small RNA seq

Gene	SNP	nt	AA Change	AA	PREP score	Cnt-1	Edited	% editing	Cnt-2	Edited	% editing	Salt-1	Edited	% editing	Salt-2	Edited	% editing
accD	CAT - TAT	229	H - Y	77	1	1	0	no editing	1	0	no editing	1	0	no editing	1	0	no editing
	TCG - TTG	617	S - L	206	0.80	7	6	0.86	5	5	1.00	8	8	1.00	0	0	nd
	ACT - ATT	764	T - I	255	0.60	6	0	no editing	6	0	no editing	4	0	no editing	15	0	no editing
	GCA - GTA	839	A - V	280	1.00	0	0	nd	1	0	no editing	0	0	nd	1	0	no editing
	TCA - TTA	1208	S - L	403	0.60	0	0	nd	2	0	no editing	6	0	no editing	4	0	no editing
atpF	CCA - CTA	92	P - L	31	0.86	0	0	nd	3	3	1.00	3	3	1.00	3	3	1.00
ccsA	TCT - TTT	563	S - F	188	0.6	0	0	nd	0	0	nd	0	0	nd	0	0	nd
	CCT - TCT	970	P - S	324	0.6	0	0	nd	0	0	nd	0	0	nd	0	0	nd
clpP	GCA - GTA	323	A - V	108	0.57	6	0	no editing	3	0	no editing	5	0	no editing	1	0	no editing
	CAC - TAC	559	H - Y	187	1	16	13	0.81	16	13	0.81	8	8	1.00	14	10	0.71
matK	TCT - TTT	11	S - F	4	0.67	15	0	no editing	19	0	no editing	12	0	no editing	24	0	no editing
	CAT - TAT	427	H - Y	143	0.86	49	0	no editing	74	0	no editing	71	0	no editing	70	0	no editing
	TCA - TTA	434	S - L	145	0.57	54	0	no editing	81	0	no editing	75	0	no editing	74	0	no editing
	CCA - TCA	571	P - S	191	0.71	1	0	no editing	0	0	nd	0	0	nd	0	0	nd
	TCT - TTT	935	S - F	312	0.57	6	0	no editing	10	0	0.00	13	0	0.00	12	1	0.08
	TCA - TTA	1022	S - L	341	0.71	6	0	no editing	8	0	no editing	10	0	no editing	6	0	no editing
	CTT - TTT	1081	L - F	361	0.57	4	0	no editing	2	0	no editing	3	0	no editing	3	0	no editing
	TCT - TTT	1235	S - F	412	0.71	3	0	no editing	4	0	no editing	1	0	no editing	1	0	no editing
	GCG - GTG	1340	A - V	447	0.86	4	0	no editing	6	0	no editing	6	0	no editing	4	0	no editing
ndhA	GCC - GTC	92	A - V	31	0.67	0	0	nd	0	0	nd	0	0	nd	0	0	nd
	GCA - GTA	137	A - V	46	1	3	0	no editing	5	0	no editing	2	0	no editing	3	0	no editing
	TCA - TTA	341	S - L	114	1	0	0	nd	1	0	no editing	0	0	no editing	0	0	nd
	TCT - TTT	1073	S - F	358	1	4	3	0.75	1	1	1.00	5	1	0.20	15	9	0.60
ndhB	CCT - CTT	74	P - L	25	1	2	0	no editing	1	0	no editing	0	0	nd	5	0	no editing

Gene	SNP	nt	AA Change	AA	PREP score	Cnt-1	Edited	% editing	Cnt-2	Edited	% editing	Salt-1	Edited	% editing	Salt-2	Edited	% editing
	TCA - TTA	149	S - L	50	1	11	6	0.55	5	4	0.80	18	6	0.33	14	5	0.36
	ACG - ATG	542	T - M	181	1	0	0	nd	1	1	1.00	1	1	1.00	0	0	nd
	CAT - TAT	586	H - Y	196	1	0	0	nd	1	1	1.00	2	2	1.00	2	0	no editing
	TCA - TTA	611	S - L	204	0.8	5	0	no editing	6	0	no editing	1	0	no editing	9	0	no editing
	CCA - CTA	737	P - L	246	1	1	1	1.00	2	2	1.00	0	0	nd	0	0	nd
	TCT - TTT	746	S - F	249	1	1	1	1.00	4	4	1.00	2	0	0.00	2	1	0.50
	TCA - TTA	830	S - L	277	1	0	0	nd	2	1	0.50	1	1	1.00	6	4	0.67
	TCA - TTA	836	S - L	279	1	0	0	nd	2	2	1.00	0	0	nd	7	6	0.86
	TCA - TTA	1112	S - L	371	1	6	4	0.67	4	4	1.00	6	5	0.83	5	3	0.60
	CAT - TAT	1255	H - Y	419	1	1	1	1.00	0	0	nd	0	0	nd	1	0	no editing
	CCC - TCC	1414	P - S	472	1	2	0	no editing	5	0	no editing	2	0	no editing	1	0	no editing
	CCA - CTA	1481	P - L	494	1	3	3	1.00	3	3	1.00	3	2	0.67	4	4	1.00
ndhD	ACG - ATG	2	T- M	1	1	1	0	0.00	1	1	1.00	0	0	nd	0	0	nd
	ACA - ATA	26	T - I	9	1	1	0	no editing	1	0	no editing	1	0	no editing	0	0	nd
	TCA - TTA	674	S - L	225	1	0	0	nd	0	0	nd	1	1	1.00	0	0	nd
	TCA - TTA	878	S - L	293	1	1	0	no editing	3	2	0.67	2	2	1.00	3	2	0.67
	TCA - TTA	1298	S - L	433	0.80	0	0	nd	2	2	1.00	0	0	nd	1	0	no editing
	CTT - TTT	1405	L - F	469	0.80	2	0	no editing	1	1	no editing	3	0	no editing	1	0	no editing
	GCT - GTT	1460	A - V	487	0.80	0	0	nd	4	4	no editing	6	0	no editing	4	0	no editing
	GCT - GTT	1475	A - V	492	0.60	0	0	nd	1	1	no editing	1	0	no editing	2	0	no editing
ndhF	CAT - TAT	13	H - Y	5	1	1	0	no editing	0	0	nd	0	0	nd	0	0	nd
	CTT - TTT	241	L - F	81	1	0	0	nd	3	0	no editing	1	0	no editing	2	0	no editing
	TCA - TTA	290	S - L	97	1	1	0	no editing	0	0	nd	1	0	no editing	0	0	nd
	CTT - TTT	388	L - F	130	1	0	0	nd	0	0	nd	1	0	no editing	0	0	nd
	CTT - TTT	586	L - F	196	0.80	1	0	no editing	3	1	0.33	2	0	no editing	1	0	no editing
	CTC - TTC	691	L - F	231	0.60	0	0	nd	0	0	nd	0	0	nd	1	0	no editing

Gene	SNP	nt	AA Change	AA	PREP score	Cnt-1	Edited	% editing	Cnt-2	Edited	% editing	Salt-1	Edited	% editing	Salt-2	Edited	% editing
	ACT - ATT	1577	T - I	526	0.60	2	0	no editing	0	0	nd	0	0	nd	1	0	no editing
ndhG	CAT - TAT	166	H - Y	56	0.80	3	0	no editing	1	0	no editing	0	0	nd	2	0	no editing
	ACA - ATA	314	T - I	105	0.80	2	0	no editing	1	0	no editing	3	0	no editing	1	0	no editing
	CAC - TAC	361	H - Y	121	1	3	0	no editing	5	0	no editing	6	0	no editing	8	0	no editing
	CCC - TCC	385	P - S	129	0.80	5	0	no editing	3	0	no editing	4	0	no editing	3	0	no editing
psaI	CAT - TAT	79	H - Y	27	1	0	0	nd	1	1	1.00	3	3	1.00	0	0	nd
psbE	CCT - TCT	214	P - S	72	1	23	21	0.91	22	20	0.91	22	20	0.91	24	24	1.00
psbF	TCT - TTT	77	S - F	26	1	8	8	1.00	10	10	1.00	14	14	1.00	7	7	1.00
rpl2	ACC - ATC	5	T - I	2	0.71	0	0	nd	0	0	nd	0	0	nd	0	0	nd
rpoA	CCC - TCC	847	P - S	283	0.71	1	0	no editing	1	0	no editing	1	0	no editing	3	0	no editing
rpoB	TCT - TTT	338	S - F	113	1	2	1	0.50	1	1	1.00	2	0	no editing	1	1	1.00
	TCA - TTA	551	S - L	184	1	0	0	nd	1	1	1.00	0	0	nd	1	0	no editing
	TCG - TTG	566	S - L	189	1	0	0	nd	3	1	0.33	1	0	no editing	2	1	0.50
	TCT - TTT	2000	S - F	667	1	1	1	1.00	0	0	nd	1	1	1.00	5	1	0.20
	ACA - ATA	2336	T - I	779	1	1	0	no editing	3	0	no editing	0	0	nd	0	0	nd
	GCT - GTT	2819	A - V	940	1	2	1	0.50	1	0	no editing	0	0	nd	0	0	nd
	GCT - GTT	3209	A - V	1070	0.57	0	0	no editing	0	0	nd	0	0	nd	1	0	no editing
rpoC1	TCA - TTA	41	S - L	14	1	0	0	nd	1	1	1.00	0	0	nd	5	0	no editing
	TCA - TTA	488	S - L	163	0.71	0	0	nd	1	0	no editing	0	0	nd	3	2	0.67
	CAC - TAC	517	H - Y	173	0.57	0	0	nd	1	0	no editing	1	0	no editing	0	0	nd
	ACG - ATG	1556	T - M	519	0.86	0	0	nd	0	0	nd	0	0	nd	1	0	no editing
rpoC2	CTT - TTT	832	L - F	278	0.86	1	0	no editing	0	0	nd	0	0	nd	0	0	nd
	CTT - TTT	1507	L - F	503	0.57	0	0	nd	0	0	nd	0	0	nd	0	0	nd
	GCA - GTA	1544	A - V	515	0.50	0	0	nd	0	0	nd	0	0	nd	0	0	nd
	CCT - TTT	1585	P - F	529	1	1	0	no editing	1	0	no editing	1	0	no editing	2	0	no editing
	CCT - TTT	1586	P - F	529	1	1	0	no editing	1	0	no editing	1	0	no editing	2	0	no editing

Gene	SNP	nt	AA Change	AA	PREP score	Cnt-1	Edited	% editing	Cnt-2	Edited	% editing	Salt-1	Edited	% editing	Salt-2	Edited	% editing
	CAT - TAT	1666	H - Y	556	0.86	1	0	no editing	0	0	nd	0	0	nd	0	0	nd
	TCG - TTG	2696	S - L	899	0.57	3	0	no editing	1	0	no editing	1	0	no editing	0	0	nd
	CAT - TAT	3004	H - Y	1002	0.57	0	0	nd	1	0	no editing	2	0	no editing	0	0	nd
	TCG - TTG	3017	S - L	1006	0.71	0	0	nd	2	0	no editing	3	0	no editing	1	0	no editing
	ACA - ATA	3284	T - I	1095	0.57	2	1	0.50	1	0	0.00	1	0	no editing	0	0	nd
	TCG - TTG	4040	S - L	1347	0.71	0	0	nd	3	0	no editing	6	0	no editing	2	0	no editing
	ACA - ATA	4046	T - I	1349	0.57	0	0	nd	1	0	no editing	3	0	no editing	3	0	no editing
	ACA - ATA	4139	T - I	1380	0.50	2	0	no editing	0	0	nd	0	0	nd	1	0	no editing
rps14	TCA - TTA	80	S - L	27	1.00	24	18	0.75	20	17	0.85	16	14	0.88	21	19	0.90
	TCG - TTG	194	S - L	65	0.71	20	1	0.05	26	1	0.04	9	1	0.11	11	1	0.09
rps16	TCA - TTA	212	S - L	71	0.83	10	9	0.90	8	6	0.75	7	4	0.57	12	9	0.75
