# Supplementary material for: Salt stress affects mRNA editing in soybean chloroplasts
Source: Genet Mol Biol. 2017 Mar 2;40(1 Suppl 1):200–8. doi: 10.1590/1678-4685-GMB-2016-0055 (PMC5452132; doi:10.1590/1678-4685-GMB-2016-0055)
Supplement: Supplementary file 2 [file 1415-4757-gmb-1678-4685-GMB-2016-0055-Suppl02.pdf]

**Table S2-** Sequences and descriptions of real time primers

| Primer | Sequence (5'-3') | Description |
|---|---|---|
| ndhA-358_Ra | CTTTTTTTTACAGTGAAAACAGTTGAA | Reverse primer for edited form |
| ndhA-358_Rg | CTTTTTTTTACAGTGAAAACAGTTGAG | Reverse primer for unedited form |
| ndhA_F | TTTCTATCGCAACAAGATGGA | Forward universal primer |
| ndhB-50_Ft | CCTAATTCTTCTTCTGATGATTGATTT | Forward primer for edited form |
| ndhB-50_Fc | CCTAATTCTTCTTCTGATGATTGATTC | Forward primer for unedited form |
| ndhB_R | CGTTGAAATTGTTCGTTTGG | Reverse universal primer |
| psbF-77_Ra | TGCTGATATTGATCCCAAAAAAA | Reverse primer for edited form |
| psbF-77_Rg | TGCTGATATTGATCCCAAAAAAG | Reverse primer for unedited form |
| psbF-77_F | TACGATGGTTGGCTGTTCAC | Forward universal primer |
| rps14-80_Ft | ATTTGATTCGCCGATCCTT | Forward primer for edited form |
| rps14-80_Fc | ATTTGATTCGCCGATCCTC | Forward primer for unedited form |
| rps14_R | GCGGTCTTCCGGTCG | Reverse universal primer |
| rps16-212_Ra | CATAAAAACACCAGCCCTTTTTA | Reverse primer for edited form |
| rps16-212_Rg | CATAAAAACACCAGCCCTTTTTG | Reverse primer for unedited form |
| rps16_F | AGCCGTTTATCGAATCGTTG | Forward universal primer |
