# Supplementary material for: Salt stress affects mRNA editing in soybean chloroplasts
Source: Genet Mol Biol. 2017 Mar 2;40(1 Suppl 1):200–8. doi: 10.1590/1678-4685-GMB-2016-0055 (PMC5452132; doi:10.1590/1678-4685-GMB-2016-0055)
Supplement: Supplementary file 3 [file 1415-4757-gmb-1678-4685-GMB-2016-0055-Suppl03.pdf]

Table S3 - Editing analyses of plastid CDS using PREP prediction and reads derived from mRNA seq

Gene	Position (nt)	PREP score	Cnt-1	% editing	Cnt-2	% editing	Salt-1	% editing	Salt-2	% editing
ndhA	**1073**	**1**	**5**	**0.60**	**7**	**0**	**11**	**0.91**	**0**	**nd**
ndhB	**149**	**1**	**5**	**1**	**0**	**nd**	**2**	**1**	**4**	**1**
psbF	**77**	**1**	**8**	**1**	**10**	**1**	**14**	**1**	**7**	**1**
rps14	**80**	**1**	**24**	**0.75**	**17**	**0.85**	**14**	**0.88**	**19**	**0.90**
rps16	**212**	**0.83**	**10**	**0.90**	**6**	**0.75**	**4**	**0.57**	**9**	**0.75**
accD	617	0.8	194	0,94	5	1	8	1	0	nd
atpF	92	0.86	36	0,97	3	1	3	1	3	1
clpP	559	1	0	nd	13	0.81	8	1	10	0.71
matK	935	0.57	0	nd	0	nd	0	nd	1	0.08
ndhB	542	1	0	nd	1	1	1	1	0	nd
	586	1	0	ne	1	1	2	1	0	nd
	737	1	1	1	2	1	0	nd	0	nd
	746	1	1	1	4	1	0	nd	1	0.50
	830	1	0	nd	1	0.50	1	1	4	0.67
	836	1	0	nd	2	1	0	nd	6	0.86
	1112	1	6	0.67	4	1	5	0.83	3	0.60
	1255	1	1	1	0	nd	0	nd	0	nd
	1481	1	3	1	3	1	2	0.67	4	1
ndhD	2	1	1	ne	1	1	0	nd	0	nd
	674	1	0	nd	0	nd	1	1	0	nd
	878	1	1	ne	2	0.67	2	1	2	0.67
	1298	0.8	0	nd	2	1	0	nd	0	nd
ndhF	586	0.8	1	ne	1	0.33	0	nd	0	nd
psaI	79	1	0	nd	1	1	3	1	0	nd
psbE	214	1	23	0.91	20	0.91	20	0.91	24	1
rpoB	338	1	2	0.50	1	1	0	nd	1	1
	551	1	0	nd	1	1	0	nd	0	nd
	566	1	0	nd	1	0.33	0	nd	1	0.50
	2000	1	1	1	0	nd	1	1.00	1	0.20
	2819	1	2	0.50	0	nd	0	nd	0	nd
rpoC1	41	1	0	nd	1	1	0	nd	0	nd
	488	0.71	0	nd	0	nd	0	nd	2	0.67
rpoC2	3284	0.57	2	0.50	0	nd	0	nd	0	nd
rps14	194	0.71	20	0.05	26	0.04	9	0.11	11	0.09
