# Supplementary material for: Salt stress affects mRNA editing in soybean chloroplasts
Source: Genet Mol Biol. 2017 Mar 2;40(1 Suppl 1):200–8. doi: 10.1590/1678-4685-GMB-2016-0055 (PMC5452132; doi:10.1590/1678-4685-GMB-2016-0055)
Supplement: Supplementary file 4 [file 1415-4757-gmb-1678-4685-GMB-2016-0055-Suppl04.pdf]

**Table S4 -** Means of RT-qPCR primer efficiency and correlation by primer

| **Primer** | **Efficiency (Mean)** | **R (Mean)** |
|---|---|---|
| *NDHA*-1073_Ra | 1.99 | 0.993 |
| *NDHA*-1073_Rg | 1.94 | 0.992 |
| *NDHB*-149_Ft | 1.91 | 0.996 |
| *NDHB*-149_Fc | 1.86 | 0.998 |
| *PSBF*-77_Ra | 2 | 0.996 |
| *PSBF*-77_Rg | 1.98 | 0.997 |
| *RPS14*-80_Ft | 1.97 | 0.993 |
| *RPS14*-80_Fc | 2 | 0.993 |
| *RPS16*-212_Ra | 1.99 | 0.994 |
| *RPS16*-212_Rg | 1.99 | 0.994 |
