# Supplementary figures and images for: Salt stress affects mRNA editing in soybean chloroplasts
Source: Genet Mol Biol. 2017 Mar 2;40(1 Suppl 1):200–8. doi: 10.1590/1678-4685-GMB-2016-0055 (PMC5452132; doi:10.1590/1678-4685-GMB-2016-0055)

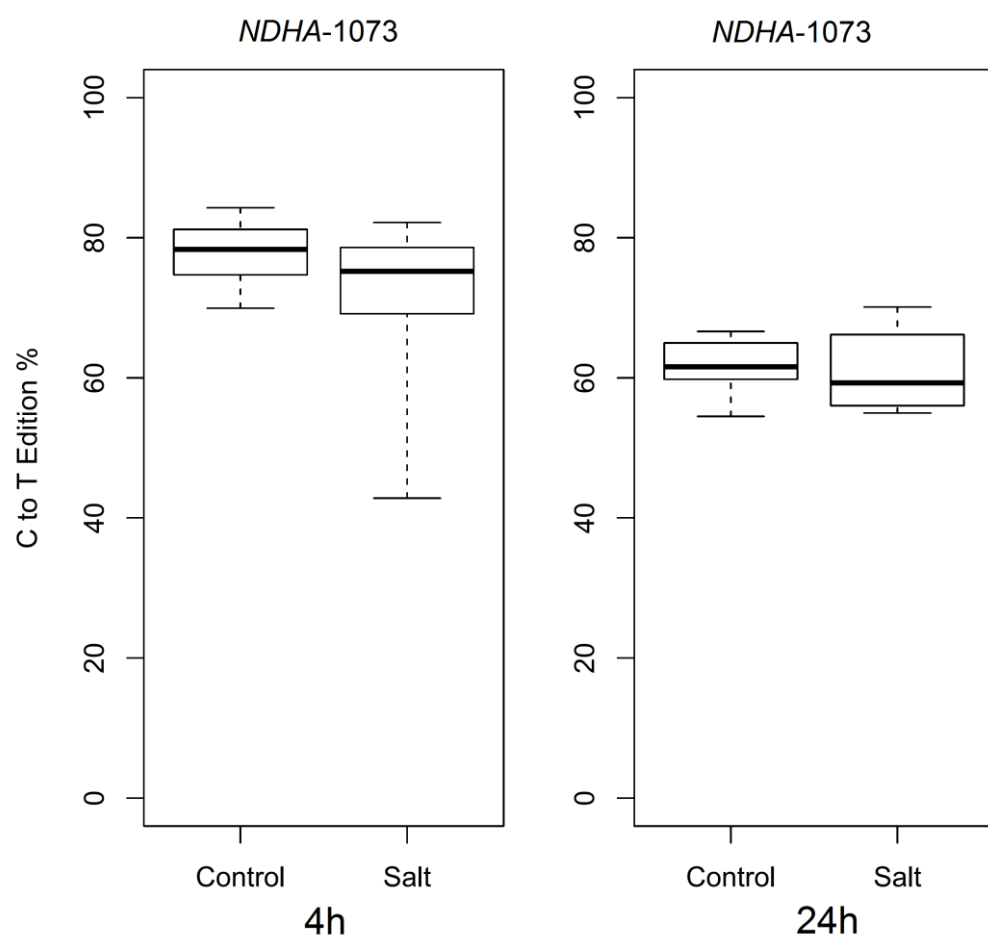

**Figure S3** - Boxplot of percentage editing of the NDHA-1073 editing site.

Supplement: Supplementary file 5 [file 1415-4757-gmb-1678-4685-GMB-2016-0055-Suppl08.pdf]

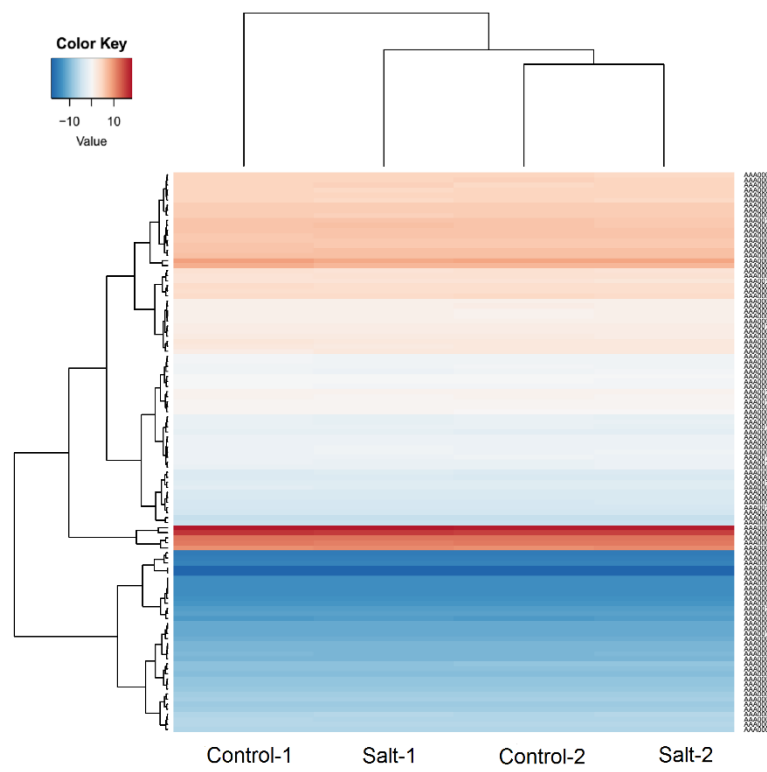

**Figure S1** - Heatmap of relative expression of plastid genes.

Supplement: Supplementary file 6 [file 1415-4757-gmb-1678-4685-GMB-2016-0055-Suppl06.pdf]
