# Supplementary material for: Salt stress affects mRNA editing in soybean chloroplasts
Source: Genet Mol Biol. 2017 Mar 2;40(1 Suppl 1):200–8. doi: 10.1590/1678-4685-GMB-2016-0055 (PMC5452132; doi:10.1590/1678-4685-GMB-2016-0055)
Supplement: Supplementary file 7 [file 1415-4757-gmb-1678-4685-GMB-2016-0055-Suppl07.pdf]

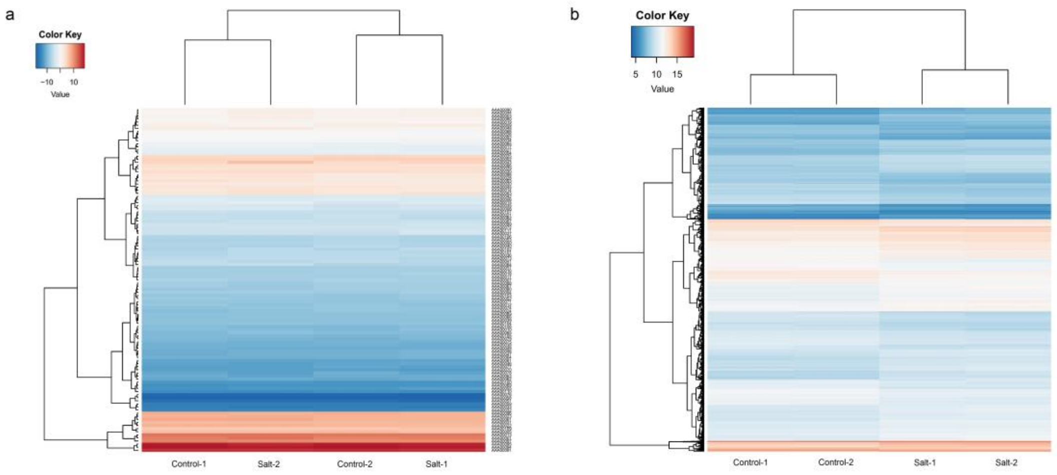

Figure S2 - Heatmap of relative expression of plastid genes and differentially expressed nuclear genes.
