# Supplementary material for: Salt stress affects mRNA editing in soybean chloroplasts
Source: Genet Mol Biol. 2017 Mar 2;40(1 Suppl 1):200–8. doi: 10.1590/1678-4685-GMB-2016-0055 (PMC5452132; doi:10.1590/1678-4685-GMB-2016-0055)
Supplement: Table S5 [file 1415-4757-gmb-1678-4685-GMB-2016-0055-Suppl05.pdf]

Table S5 - Identification of chloroplast genes in heatmap.

Gene	Identifier
GlmaCt001 trnH-GUG	AAA00001
GlmaCp001 psbA	AAA00002
GlmaCt002 trnK-UUU	AAA00003
GlmaCp002 matK	AAA00004
GlmaCp003 rbcL	AAA00005
GlmaCp004 atpB	AAA00006
GlmaCp005 atpE	AAA00007
GlmaCt003 trnM-CAU	AAA00008
GlmaCt004 trnV-UAC	AAA00009
GlmaCp006 ndhC	AAA00010
GlmaCp007 ndhK	AAA00011
GlmaCp008 ndhJ	AAA00012
GlmaCt005 trnF-GAA	AAA00013
GlmaCt006 trnL-UAA	AAA00014
GlmaCt007 trnT-UGU	AAA00015
GlmaCp009 rps4	AAA00016
GlmaCt008 trnS-GGA	AAA00017
GlmaCp010 ycf3	AAA00018
GlmaCp011 psaA	AAA00019
GlmaCp012 psaB	AAA00020
GlmaCp013 rps14	AAA00021
GlmaCt009 trnfM-CAU	AAA00022
GlmaCt010 trnG-GCC	AAA00023
GlmaCp014 psbZ	AAA00024
GlmaCt011 trnS-UGA	AAA00025
GlmaCp015 psbC	AAA00026
GlmaCp016 psbD	AAA00027
GlmaCt012 trnT-GGU	AAA00028
GlmaCt013 trnE-UUC	AAA00029
GlmaCt014 trnY-GUA	AAA00030
GlmaCt015 trnD-GUC	AAA00031
GlmaCp017 psbM	AAA00032
GlmaCp018 petN	AAA00033
GlmaCt016 trnC-GCA	AAA00034
GlmaCp019 rpoB	AAA00035
GlmaCp020 rpoC1	AAA00036
GlmaCp021 rpoC2	AAA00037
GlmaCp022 rps2	AAA00038
GlmaCp023 atpI	AAA00039
GlmaCp024 atpH	AAA00040
GlmaCp025 atpF	AAA00041
GlmaCp026 atpA	AAA00042
GlmaCt017 trnR-UCU	AAA00043
GlmaCt018 trnG-UCC	AAA00044

Gene	Identifier
GlmaCt019 trnS-GCU	AAA00045
GlmaCp027 psbI	AAA00046
GlmaCp028 psbK	AAA00047
GlmaCt020 trnQ-UUG	AAA00048
GlmaCp029 rps16	AAA00049
GlmaCp030 accD	AAA00050
GlmaCp031 psaI	AAA00051
GlmaCp032 cemA	AAA00052
GlmaCp033 petA	AAA00053
GlmaCp034 psbJ	AAA00054
GlmaCp035 psbL	AAA00055
GlmaCp036 psbF	AAA00056
GlmaCp037 psbE	AAA00057
GlmaCp038 petL	AAA00058
GlmaCp039 petG	AAA00059
GlmaCt021 trnW-CCA	AAA00060
GlmaCt022 trnP-UGG	AAA00061
GlmaCp040 psaJ	AAA00062
GlmaCp041 rpl33	AAA00063
GlmaCp042 rps18	AAA00064
GlmaCp043 rpl20	AAA00065
GlmaCp044 rps12	AAA00066
GlmaCp046 clpP	AAA00067
GlmaCp047 psbB	AAA00068
GlmaCp048 psbT	AAA00069
GlmaCp049 psbN	AAA00070
GlmaCp050 psbH	AAA00071
GlmaCp051 petB	AAA00072
GlmaCp052 petD	AAA00073
GlmaCp053 rpoA	AAA00074
GlmaCp054 rps11	AAA00075
GlmaCp055 rpl36	AAA00076
GlmaCp056 rps8	AAA00077
GlmaCp057 rpl14	AAA00078
GlmaCp058 rpl16	AAA00079
GlmaCp059 rps3	AAA00080
GlmaCp060 rps19	AAA00081
GlmaCp061 rpl2	AAA00082
GlmaCp062 rpl23	AAA00083
GlmaCt023 trnI-CAU	AAA00084
GlmaCp063 ycf2	AAA00085
GlmaCt024 trnL-CAA	AAA00086
GlmaCp064 ndhB	AAA00087
GlmaCp065 rps7	AAA00088
GlmaCp045 rps12	AAA00089

**Gene**	**Identifier**
GlmaCt025 trnV-GAC	AAA00090
GlmaCr001 rrn16	AAA00091
GlmaCt026 trnI-GAU	AAA00092
GlmaCt027 trnA-UGC	AAA00093
GlmaCr002 rrn23	AAA00094
GlmaCr003 rrn4.5	AAA00095
GlmaCr004 rrn5	AAA00096
GlmaCt028 trnR-ACG	AAA00097
GlmaCt029 trnN-GUU	AAA00098
GlmaCp066 ycf1	AAA00099
GlmaCp067 rps15	AAA00100
GlmaCp068 ndhH	AAA00101
GlmaCp069 ndhA	AAA00102
GlmaCp070 ndhI	AAA00103
GlmaCp071 ndhG	AAA00104
GlmaCp072 ndhE	AAA00105
GlmaCp073 psaC	AAA00106
GlmaCp074 ndhD	AAA00107
GlmaCp075 ccsA	AAA00108
GlmaCt030 trnL-UAG	AAA00109
GlmaCp076 rpl32	AAA00110
GlmaCp077 ndhF	AAA00111
